# Agile roadmap for application‐driven Multi‐UAV networks: The case of COVID‐19

**DOI:** 10.1049/ntw2.12040

**Published:** 2022-08-23

**Authors:** Laila Abouzaid, Halima Elbiaze, Essaid Sabir

**Affiliations:** ^1^ TicLab International University of Rabat Rabat Morocco; ^2^ Department of Computer Science University of Quebec at Montreal (UQAM) Montreal Quebec Canada; ^3^ NEST Research Group LRI Lab ENSEM Hassan II University of Casablanca Casablanca Morocco

## Abstract

Drones, also known as Unmanned Aerial Vehicles (UAVs), are about to bring drastic transformations to our world and daily lives. News thinking and efficient deployment are required to boost the adoption of UAV‐augmented commercial/civil applications. Yet, network service providers are still facing several design challenges of UAV‐assisted application, due to lack of a roadmap allowing to meet the target service level agreement requirements. In this paper, we propose a complete framework for the UAV as a service paradigm, integrating all the actors/stakeholders contributing to the UAV‐augmented service, and draw their interactions using data/service/money flows. Next, we instantiate our framework on the COVID‐19‐like pandemics, and discuss how to use it force social distancing, spray disinfectants, broadcast messages, deliver medical supplies and enhance surveillance. Computer simulation provide insights on how to set the multi‐UAV network to combat COVID‐19.

## 1 INTRODUCTION

Unmanned Areal Vehicles (UAVs) open up new ways and create leverage for various stunning applications over the world. They are being massively adopted in civil and military domains. Among the benefits this technology offers, one mentions of a low cost, ability to perform complex tasks reducing the risk of human life and a flexible deployment in remote and inaccessible areas. From target recognition and surveillance to electronic attack in military missions, drones have evolved towards a wide range of applications, such as Search and Rescue (SAR), road traffic, tower inspection, etc.. [1, 2]. However, the unprecedented growth of UAV applications has resulted in numerous challenges to be solved, including on‐the‐fly deployment, optimal 3D‐placement, path planning, energy‐efficiency [3] and other challenges.

From a drone‐assisted application perspective, the stringent requirements in terms of Quality of Service (QoS), Quality of Experience (QoE), mobility, dynamic network topology, traffic demand, real‐time, reliability, energy constraints, etc., call both academia and industry professionals to join efforts, and build by design application instead of trying to adapt existing applications. Network service providers are aware that such a paradigm shift implies the need for a whole new network (re)design in order to support hybrid, context‐adaptive services and mission‐critical tasks efficiently and even fundamentally. Ultimately, it is essential to develop innovative UAV applications that are able to meet the Service Level Agreement (SLA) (in terms of data rate, reliability, latency, jitter, data freshness, data correctness, cost, etc.), given some knowledge on the target environment. In other words, deploying a UAV application using a generic model and/or without considering application specific requirements turns to be a lost battle. Therefore, designing a UAV‐assisted application network must be at the top of any UAV service provider objective's list, as the UAV‐network application's characteristics and requirements influence the design in many ways. The UAV service provider needs to efficiently size, plan and strategically deploy its UAV network with tweaked internal parameters settings, according to predictable service levels imposed by each application in order to provide the best experience and stay ahead of this very lucrative market.

In line with this vision, we propose a framework for UAV‐as‐a‐service (UAVaaS), integrating the actors/stakeholders contributing to UAV application design and draw their interactions and functionalities as well. The ultimate aim of this paper is to shed light to UAV design planners' missions. Especially, we provide insights on fine‐tuning parameter settings for a UAV network to keep up with the application needs and SLA specifications. As a use‐case, we exhibit a simple projection of our framework on COVID‐19‐like pandemics. Computer simulation shows how our scheme could efficiently provide a UAV solution allowing to monitor, enforce lockdown, track, control and handle the pandemics outbreak through the video surveillance application, which might be of precious help to decision‐makers, health authorities and insurance companies.

In Section 2, we present the related work relating to the challenges associated with efficiently deploying drones. In Section 3, the application requirement while designing the UAV‐based application is discussed. Then, we present our UAVaaS framework in Section 4. Section 5 exhibits how our framework can be used to design powerful applications to combat Corona‐Virus‐like pandemics and we simulate a multi‐UAV network implementing our framework, and open research issues are presented in Section 6. Conclusions are drawn in Section 7.

## 2 RELATED WORK

Drone‐based application providers need expertise in a number of fields including geography/environment, model/network design, wireless networking, technology, drone route‐planning and so on. Several studies that address each of these issue areas/challenges are discussed separately in the literature.

A 3D deployment requires fundamental planning includinng quality of service (throughput, latency, coverage, etc.) maximization, and density/scalability support. Deploying UAVs optimally relies on a variety of parameters, including ground target location, geographic environment, and the strength of the A2G links. Also, the simultaneous deployment of multiple UAVs is increasingly challenging due to the effect of inter‐cell interference on system efficiency. For example, the authors in [4] propose a heuristic algorithm based on particle swarm optimization. It aims to find optimally the minimum number of UAVs to be deployed. Also, the optimal placement of UAV that maximises the throughput is discussed in [5]. The authors in [5] used heuristic and approximation algorithms to optimally deploy UAV with respect to its associated users within the transmission range. In [6], the authors investigated the optimal UAV Base Station (UAV‐BS) altitude for a single and two UAV‐BS while offering a good coverage area and a low energy consumption. Also, the authors study the impact of the distance that separates the two‐UAV on the coverage zone, and they find the optimal distance between the drones that ensures a maximal covering. While deploying multi‐UAVs within a given area, the presence of co‐channel interference affects the UAV communication performance. The work [7] presents a multi‐UAVs strategy for an optimum deployment in the presence of co‐channel interference for two scenarios. It is a symmetric scenario when all the UAVs have the same height and an asymmetric scenario when the drone is located at a different height. An optimum distance separated UAVs in order to mitigate the co‐channel and maximise the coverage performance is obtained for suburban and urban environments. Moreover, the 3D deployment of the drone by minimising the energy consumption is discussed in [8, 9]. The authors in [8] propose an optimal placement algorithm for the drone by jointly maximising the number of covered users on the ground and minimising their transmit power. Using circle packing theory, the authors in [9] propose an approach allowing the UAV to transmit with a minimum power, which maximises the coverage by avoiding the overlap between UAVs. They have shown that the UAVs’ placement is determined based on the gain/beam‐width of the drone's directional antennas and the number of UAVs. Additionally, the UAVs plays a vital role in IoT communication, which is formed of devices with limited battery. Due to the limited battery, the IoT devices cannot send over a long distance; the UAVs operate as aerial base stations to collect data from the ground IoT devices. For that, the work [10] proposes a novel framework aiming to optimise the 3D deployment with the objective of being energy‐efficient from IoT nodes on the ground.

Unmanned Aerial Vehicle path planning is another major challenge facing UAV integrated networks. Literature has devoted considerable attention to optimising UAV trajectory in a variety of applications. As discussed in [11], the authors define and illustrate the different applications supported by a Flying Ad Hoc Network (FANET) and identify appropriate mobility models for each application and scenario goal such as SAR, Forest fire detection, Traffic, and urban monitoring, among other applications. For example, Path‐Planned Mobility Models can be suitable for several applications (e.g: SAR, Agricultural management, delivery of goods…) since it holds a different movement pattern. Furthermore, the authors in [12] analysed the impact of various mobility patterns (e.g. Tractor, Angular, Square, Circular) on UAV performance such as coverage, Time Efficiency, and Utilization, etc.. The authors present a trade‐off between maximising the covered nodes while minimising the time efficiency allowing to choose the suitable mobility pattern through the finding performance results. In [13], the authors maximise the throughput of a UAV mobile relay while jointly optimising trajectory and source/destination nodes that transmit power.

Further, an understanding of wireless communication propagation is crucial to the long‐term success of interoperable UAV networks. A considerable amount of effort has been devoted to channel measurements and modelling, so as to characterise the ground‐to‐air and air‐to‐air communication channels. The propagation path loss, fading and delay spread are provided for A2G characterisation in [14], by using a mix of computer simulations and measurement results. In [15], the authors provide both mathematical and simulation models for studying the effect of rain, cloud and gaseous absorption attenuation on high height A2G connection. As outlined in [16], positioning UAVs properly may experience the best A2G channel quality (greater probability of LoS) as against fixed terrestrial base stations. A propagation model in an urban area for mobile communications through HAPs is presented in [16]. An empirical propagation prediction model is described as a function of the elevation angle. Mainly the authors in [16] derive the probability of LoS between the High‐Altitude Platform (HAP) and ground users by considering the Non‐Line‐of sight (NLoS). The authors in [17] derived the probability of LoS connection for the A2G link by considering the average altitude of buildings in urban environments. The presented works give some insight into a generic model's characterisation for A2G communication.

For any UAV‐assisted wireless communication system deployed or to be deployed, understanding a network performance before and after deployment is required, from network planning and design phase to network survivability. Obviously, a crucial performance analysis must be performed while designing UAV‐enabled wireless communication. It can be accomplished through computer‐based simulations, theoretical analysis, or an experimental field test. Performance analysis for UAV‐based systems have recently attracted much attention. The existing works analyse the performance of UAV‐based communication for different purposes such as relay or ad hoc network and from different spatial locations of the aerial/ground nodes. For UAV coverage analysis, reference [18] used a stochastic geometric method to provide an analytic expression for coverage probability where different drones manage ground users. The work in [18] analysed the coverage as a function of UAV parameters (e.g., altitude, antenna bandwidth) and under Nakagami‐m fading channel; they demonstrate how the coverage can be maximised for a given value of UAV parameters. The coverage metric for a UAV network‐served downlink and Device‐to‐Device (D2D) users is analysed in [19]. First, the authors derived the downlink coverage probability and system rate for downlink users and D2D devices. Then the impact of D2D density and UAV altitude on the overall performance system, such as coverage, system rate and number of stops, was analysed. Also, the authors in [20] provided the analytical study for achievable coverage and throughput by an aerial base station. In order to find the optimal altitude that maximises the coverage, the proposed framework was implemented in a realistic urban environment with architectural statistics based on the recommended parameters by the ITU. Nevertheless, to successfully deploy a UAV network, it is important to guarantee the reliability of the UAV link. For example, the authors in [21] derived the outage probability depending on altitude and elevation angle in both communication modes: direct A2G and A2A communication using a relaying.

## 3 DEPLOYING APPLICATIONS ON THE AIR

The never‐seen popularity of UAVs in various domains has triggered a surge deployment of novel applications while meeting agreed SLA specifications. However, the designer still needs to deal with numerous interdisciplinary challenges. Yet, those challenges are strongly specific to each single application, based on the application context such as the target environments (urban, rural, etc.), mobility pattern, QoS/QoE level, communication range, network density, etc. In this subsection, we summarise the important features considered whilst designing an application.

### 3.1 UAV typology

UAVs can be categorised according to several criteria such as altitude, wings shape, size and endurance capability. In terms of altitude, there are two existing categories: HAP/Low‐Altitude Platform (LAP). A HAP provides ubiquitous Internet/mobile services to a wide geographical area at a height above 17 km, see Google's Project Loon (https://loon.com/). A LAP operates at a height below few kilometres, can move fast and it is flexible. Furthermore, LAPs can substantially extend broadband access between ground‐air by establishing a Line‐of‐Sight (LoS). Visual line‐of‐sight and Beyond Visual Line‐of‐Sight are the most used drones for urban low‐altitude applications such as those used in response to the COVID‐19‐pandemic.* Besides, UAV types are also classified according to either their wing type (fixed/rotary) or their size [22, 23]. Consequently, selecting the appropriate UAV for a specific application/mission suggests to consider other features such as endurance, payload etc. is paramount.

### 3.2 Single‐UAV VS. Multiple‐UAV

Deploying a single‐UAV or UAV‐swarm (multi‐UAV) for a desired application is one of the basic challenges facing the UAV designers. A single UAV provides restricted operational missions while deploying a multi‐UAV network eases complex mission’s completion by exploiting coordination and cooperation between UAVs. A performance comparison of single and multi‐UAV systems according to multiple features, including, coverage [24], topology, security, survivability and cost, is investigated in [25]. In terms of coverage, the authors show that single‐UAV is inappropriate for wide regions. Meanwhile, multi‐UAV can complete missions with a higher chance of coverage, extended survivability and better performance. From security perspective, a drone requires only one link making it less vulnerable to attacks compared to multi‐UAV with multiple vulnerabilities [25]. Deploying an application on the air requires a careful design to identify the appropriate topology that can meet the use‐case requirements [23].

### 3.3 Federating communication within a UAV‐swarm

A robust communication architecture is a fundamental step in the design of UAV‐swarming. It describes how data are exchanged between the ground control station (GCS) and drones or between drones. Unmanned Aerial Vehicle communication subsystem can be either centralised or decentralised. In the centralised architecture, each drone is directly attached to GCS, that handles every single operation. Whereas, the decentralised fashion allows UAV‐to‐UAV links and infrastructure less ad hoc communications. Under decentralised architecture, one cites three schemes:

– **UAV‐Swarm:** UAVs communicate in an ad hoc manner without the involvement of a GCS. Each UAV act as a relay, means that the data goes along multiple hops until reaching the final destination. This scheme is suitable for a group of homogeneous drones;
– **Multi‐Swarm:** Each swarm consists of an ad hoc network with its own UAV‐backbone connected to the GCS. Intra‐swarm communications do not need the GCS, while inter‐swarm communications are met through the GCS;
– **Hierarchical Multi‐Swarm:** This topology defines three layers: UAV swarms with UAV‐to‐UAV communications; inter‐swarm communications composed of UAV gateways associated to each swarm, and the third layer includes the closest UAV‐group that is connected to the GCS through its backbone.

The choice of the topology is closely related to the application/mission constraints/purposes. Indeed, centralised topology fits with applications designed for small number of UAV‐swarms and for merely simple missions. Unfortunately, centralised solution might suffer from scalability, robustness and coverage issues. Decentralised topology solves those issues at the cost of an extra Capital Expenditure and Operational Expenditure and additional complexity.

### 3.4 Wireless communication technologies

Selecting the appropriate wireless technology for UAV‐communications is critical for meeting the application requirements and fulfils its SLA. Wireless communication technologies can be split into: Short‐range communications including Wi‐Fi, Zigbee, etc.; and long‐range communications usually requiring a dedicated infrastructure and specific licence, including 4 G/5G, LoRa, Sigfox, NB‐IoT, satellite, etc. A comprehensive study in [26] exhibits existing wireless technologies available for UAVs in terms of data type, node density, mobility, communication range, spectrum, target environment, etc.. Furthermore, the wireless technologies used for UAV communications can operate over licenced or/and unlicensed bands, which might require a specific licence from the regulatory authorities.

### 3.5 Mobility pattern

High mobility and extreme agility are two of the most remarkable features of UAV‐swarm. They drastically impact the UAV network performance especially the routing efficiency. Prior to the deployment stage, the designer needs to emulate a realistic FANET environment. Thus, he/she has to implement a mobility model imitating the motion of flying vehicles. Mobility model defines the UAVs trajectories and how their location, direction, speed and acceleration change over time [27]. The main existing mobility models for multi‐UAV systems are: Pure Randomized Mobility Models, Time‐Dependent Mobility Models, Path‐Planned Mobility Models, Group Mobility Models and Topology‐Control–Based Mobility Models.

The authors of [11, 28], classify, illustrate applications supported by FANET and list the appropriate mobility models for each application/mission such as SAR, forest fire detection, traffic and urban monitoring among other applications.

### 3.6 Routing protocol

Routing is a vital component in reliable end‐to‐end data transmission, as it provides a map describing how the drones can communicate mutually. Most of the traditional ad hoc routing protocols are inappropriate, due to the drone's specific features namely, high speed, topology changes, etc. A huge research effort has been spent to fully/partially adapt existing routing protocols for FANETs. Luckily, many of them have been successfully ported to the UAV‐environment. There are five categories of routing protocols: static protocol, proactive protocols, reactive routing protocol, hybrid protocols and position/geographic‐based protocols. It is worth noting that the static routing is not scalable and does not fit with multi‐UAV networks, with dynamic topology, since the routing table cannot be modified and uploaded during flight. Meanwhile, the position‐based routing protocols use GPS to locate UAV nodes, which is useful when the topology changes frequently. We invite the reader to check [29] for comprehensive comparisons of the fundamental routing protocols in UAV networks.

## 4 DESIGNING UAV‐ASSISTED APPLICATIONS

### 4.1 Network application by design

Designing a UAV‐Network is intended to forecast, comprehend, evaluate, measure and scope out realistic network implementation over time. The design phase must be executed to make sure the application works properly and the SLA is fulfiled. Network operators are striving for different ways to maintain well‐designed UAV‐based application systems taking into account the metrics cited above. However, by having comprehensive application requirements, there is a need to determine a set of physical/Medium Acccess Control parameters to satisfy the SLA specifications, support the traffic offered and thereby overcome traffic congestion. These parameters include altitude, antenna aperture angle, angular velocity, speed, transmit power, etc. which impact the global network performance. It is, therefore, important to evaluate the performance metrics to have insights on the setting parameters. The main factors that describe the motivation for designing the UAV‐network are:

– **Network performance Assessment**: Undoubtedly, guaranteeing an efficient UAV network anytime and anywhere is the ultimate goal for any service provider and/or user. Therefore, evaluating the performance metrics before implementation is the first step to do, and a safe way to
– **Coverage Area**: It varies with the application needs and the usage/user context. By setting the control parameters (i.e., altitude, aperture angle, transmit power, speed, etc.) properly, and having some knowledge on the channel variabilities (i.e., fading, path‐loss, interference, etc.), the coverage area can be strategically adjusted as the need arises;
– **Overseeing the Network**: UAV‐Network designs using mathematical modelling [30] and/or computer simulation, aim to give a reliable understanding of UAV‐based application behaviour. After the simulation phase, the network monitoring becomes possible to view and adjust, if needed, the parameter setting to meet the application requirements.

### 4.2 UAV as a service framework: stakeholders

The UAV‐assisted application provider must follow all the processes and development steps of the application to meet desired SLA by the end‐user. However, sharing information and services among numerous actors responsible of several processes is not optimal as this results in delays and additional cost in the planning phase. The UAV as a Service concept comes to steer clear of such a problem. Indeed, UAVaaS concept is a complete roadmap that aims to describe the operations of developing, deploying and exploiting a UAV application, which offers a complete vision of the whole process. Namely, it delivers the tools, administrative steps, technologies and materials to the future application/service provider. UAVaaS integrates all the actors, also called parties, involved to guarantee a successful UAV‐assisted application. Hereafter, we describe the role of each involved party.

#### 4.2.1 End‐user

It is the entity that uses the service made available through the UAV network. It possibly includes IoT devices (cameras, sensors, smart‐metres, etc.) and humans using a smart device (mobile phone, tablet, wearable device, etc.). The end‐user has to use the application after it has been fully deployed and properly tested. Due to a human subjective perception, the end‐user may claim improving the QoS/QoE when the service is not delivered properly.

#### 4.2.2 Application service provider (ASP)

This is the customer who intends to deliver the UAV‐assisted application and its related services to end‐users across a drone network. ASP may be telecommunication companies that offer connectivity to their users, a business enterprise that carries out its own missions (surveillance, construction, etc.), an individual person seeking to optimise his/her exploitation, a non‐profit organization or governmental organizations that offer services and support to citizens (remote healthcare, disaster management, etc.). In essence, the ASP is tasked with overseeing the end‐user satisfaction while building a useful added‐value business.

#### 4.2.3 National aviation authority (NAA)

NAA is an organization or a collaborative institute. It provides the rules, administrative aspects, technical guidelines and utilization policies that describe how a drone might fly safely and legally. Thereby, it is obligatory to request an airspace authorisation to fly over a specific area or over the national territory. Moreover, NAA is also tasked to assign the frequency band available depending on communication technology to be used by the UAV application.

#### 4.2.4 Application requirement data repository (ARDR)

ARDR is the data library or the archive. Its role is data storage so that the information can be efficiently mined and used for experiments, simulation and/or analytical purposes. This repository may include: (i) UAV application requirements in terms of QoS parameters, namely, throughput, latency, packet loss and jitter for multiple traffic classes (video, image, audio, data); (ii) log files on the service exceptions and failures; (iii) target devices' features (IoT sensors, handsets, etc.); and (iv) assessment of the backbone’s performance that is used to store the data. The main task related to this entity is to collect application requirements recommended by authorities and agencies, such as the International Telecommunication Union [31]. Therefore, the data collected is stored in the repositories to be mined by the planner design, and checked afterwards if the SLA is met.

#### 4.2.5 User data repository (UDR)

This entity pertains to storage and backup operations in/from the cloud, over data collected from end‐users. Thus, ensuring a trusted data governance is paramount. An accurately administered data repository may significantly coerce data protection and cyber security of sensitive data. Even if data repositories are managed by a trustworthy entity such as government or health sector agencies, privacy commissioners need to be allowed access if investigation/audit is required. Moreover, minimum data retention is another critical issue for UDR as collected data should not be preserved beyond the application/mission lifetime. Once the envisioned purpose of the application is achieved, all the related data in UDR should be destroyed.

#### 4.2.6 Flying infrastructure provider (FIP)

A good understanding of the target area is paramount, while developing the UAV‐assisted application. The propagation environment (urban, suburban, rural, forest, mine, etc.), the propagation medium (dry or moist air), and surface configuration (buildings, reliefs, hills, trees, etc.) are the set of terrain data that FIP must retrieve. Having sufficient knowledge on terrain features provides important insights on a wireless radio channel, which reveals the channel properties thereby identifying the appropriate infrastructure. The FIP can either build her decision based on analytical models or empirical/statistical data, taking into account budget, available resources (software and hardware) and crew skills. In addition to being expensive, it is understood that empirical methods might sometimes be unfeasible in hard‐to‐reach environments. Whereas, analytical methods relying on the channel’s estimation/simulation approaches (deterministic/stochastic) aided by some knowledge on the channel features, buildings and obstructions parameters, seem to provide better accuracy‐cost trade‐offs [32, 33].

#### 4.2.7 Design planner (DP)

This entity can be either an individual or an organization. Its core role is to encompass the environment design, topological design, UAV‐network analysis/assessment, etc., to guarantee that a new delivered UAV‐assisted application keeps up with the needs of customer/users.

- *Environment Design:* This step incorporates the terrain data (provided by FIP) into the simulation environment (NS2/NS3, Optimized Network Engineering Tools (OPNET), Omnet+, etc.). Terrain data includes signal attenuation, shadowing and path‐loss between each UAV and the other UAV/ground nodes on a given spectrum, bandwidth and radio wave propagation model;
- *Topological Design:* This step places the nodes (UAV, ground IoT, terrestrial base station, etc.) in the space and sets the drones’ trajectories. The mobility pattern recommended for a specific application is calculated based on the service SLA and the target area;
- *Layer/Slice Model Design*: This step defines the layers/slices of the application‐driven UAV swarm (OSI model, TCP/IP, LoRaWAN stack, etc.);
- *UAV‐network Analysis:* When the model/simulation parameters are set (i.e., environment, topology and layers) the design planner is now able to generate typical scenarios for different settings. All these scenarios are used to determine the optimum parameters that meet the SLA specifications for the desired application. Amongst others, the output parameters to be provided include: transmit power, altitude, antenna aperture, speed, trajectory, density of drones, etc.

#### 4.2.8 UAV deployment ground crew (UAV‐DGC)

The crew of UAV‐DGC is committed to prepare the tools to be used during the implementation phase (drones, sensors, ground station, etc.). Incorporating the optimal setting, the UAV swarm is implemented in the area of interest. This actor is also responsible for monitoring the UAV’s fleet during take‐off, flight and landing to guarantee a safe flight, and avoid/handle any hypothetical accidents. This entity in turn offers an implementation with full materials/service components.

#### 4.2.9 UAVaaS coordinator

This actor is at the heart of the UAVaaS framework. It is engaged to accompany and assist the internal and external parties, with the aim of creating synergy among these actors to ensuring a flawless exchange of incoming and outgoing information. All the data and services' flows pass through the UAVaaS coordinator.

### 4.3 UAVaaS flows

**FIGURE 1:**
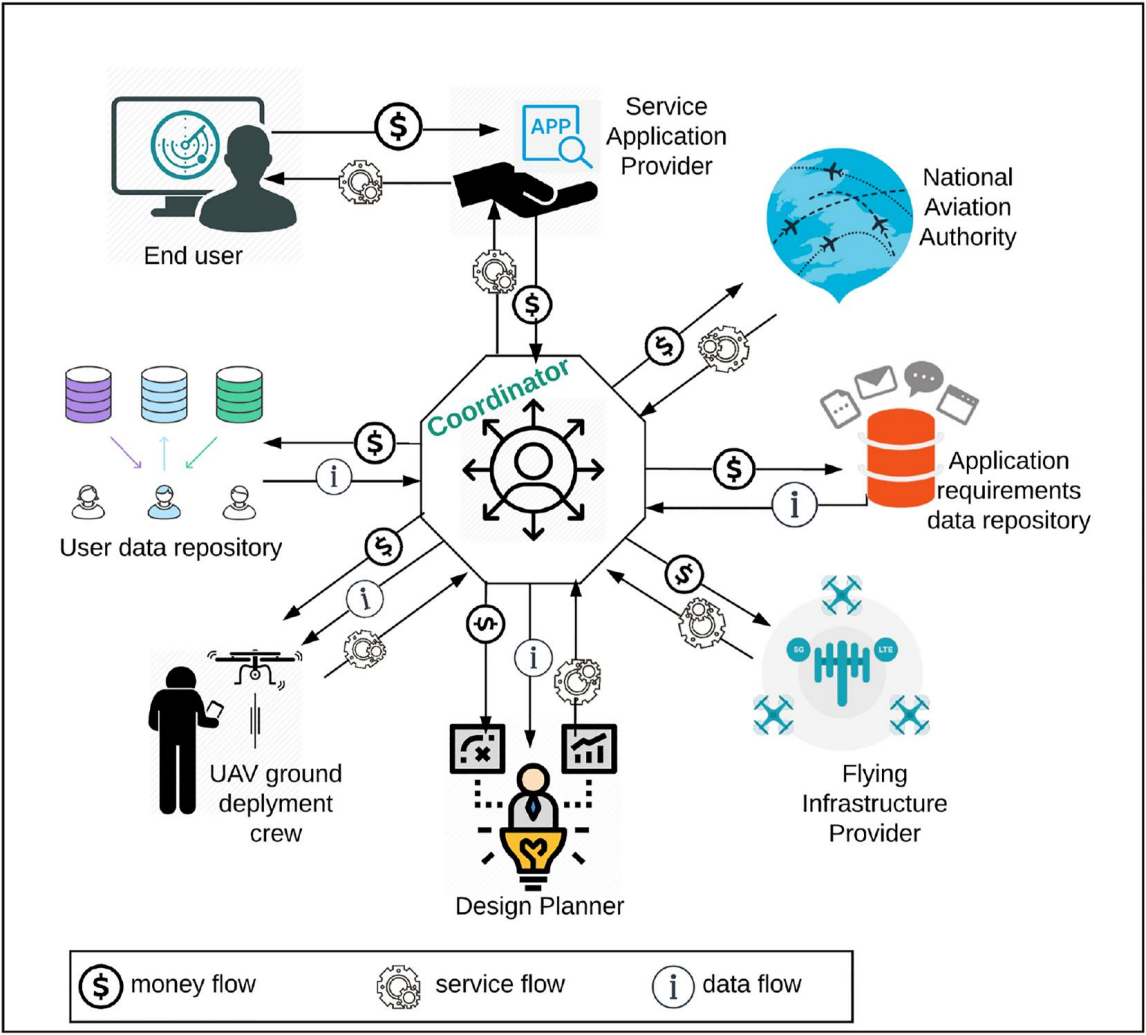
UAV‐assisted application design and business model

Cross interactions involve three types of flows: service/data/money flows. The data flow shows the streams of information circulating between any source‐destination pair. Data is mostly digital, and is collected through observation, measurement, simulation or generated by devices. The service flow refers to the relationship between a provider and a customer. The provider may offer services, softwares, infrastructures, platforms, materials, etc. Regarding the money flow, it defines how cash is moving in and out of the UAVaaS ecosystem, who pays who and for what. Figure 1 shows the interaction/flow between each source‐destination pair. These interactions are sketched as follows:

1. The ASP asks the UAVaaS coordinator to identify the application type and the sale process of the service. The UAVaaS returns information about the billing policies and progress status of the selected service. The billing policy varies from one service to another, based on the number and type of drones, the software/hardware used for storage and/or data communication and on‐board sensors;
2. The UAVaaS Coordinator requests a drone flight authorisation from the NAA. The latter offers an exclusive authorisation and/or provides the required spectrum;
3. The ARDR identifies the UAV‐application requirement in terms of communication aspects. This information in turn must be shared with the UAVaaS to coordinate all the other aspects;
4. The FIP is expected to generate real/simulated data about the terrain where the application will be deployed. This is merely helpful in order to identify the appropriate flying infrastructure;
5. The UAVaaS coordinator forwards the collected data to the design planner, to efficiently plan the application‐based UAV network;
6. Parameters collected by the design planner are forwarded to the UAV‐DGC. The design planner and the UAV‐DGC should communicate in an explicit way to assess the whole solution and constantly revise the design when needed. Besides, the ground crew has to constantly inform the coordinator about the implementation progress;
7. The UDR module offers a storage service and adapted equipment to the UAVaaS coordinator;
8. The end‐user and the ASP need to share the experience while using the service. Certainly, the money flow is ruled by the adopted business model. Usually, end‐users aren't asked to directly pay the ASP but this could be taken in charge by tier‐parts, in the same way video streaming is freely available via advertisement.

## 5 UAV‐AUGMENTED SURVEILLANCE AND WARNING FOR COVID‐19‐LIKE PANDEMICS

Public and private organizations are working hard to find creative ways to minimise the effects of the COVID‐19 pandemic. UAV‐based solutions present a versatile tool for managing this health catastrophe while reducing the risk of the virus transmission especially among front‐line workers.

### 5.1 UAV‐augmented surveillance‐warning requirements

**FIGURE 2:**
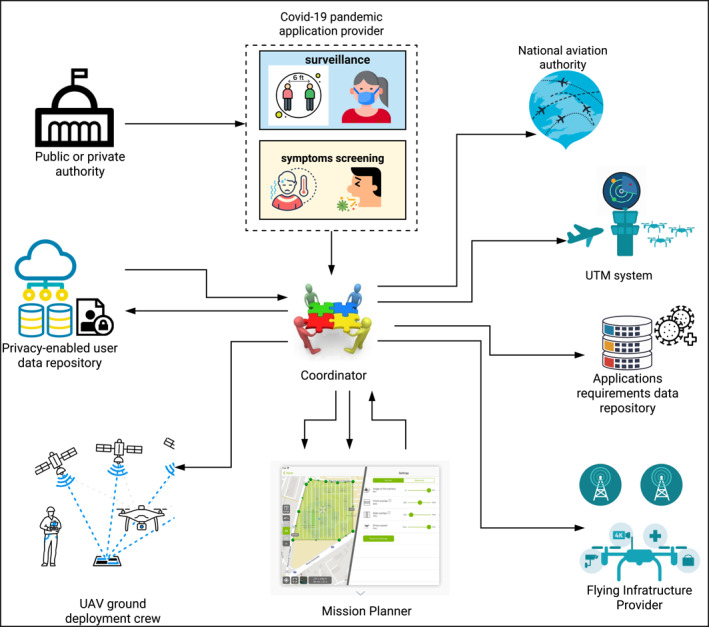
UAV network design for COVID‐19 surveillance

Countless applications where drones are involved are mentioned in the introduction section. Multiple applications deploy drones in the sky with the task of collecting information describing what is happening in the area of interest. The collected information may come in different forms: video streaming, images, maps, data, etc.. Recently, surveillance using UAVs has become the most controversial use, whether for tracking humans in a natural disaster or terrorist attacks or road traffic applications or other purposes. In response to the COVID‐19 pandemic, robotic and/or UAV‐based applications are used to enforce social distancing, spray disinfectants, broadcast messages, deliver medical supplies and enhance surveillance. The use case envisaged in this work is inspired by this period. For this purpose, we consider a UAV‐swarm deployed in an area. Each drone is equipped with cameras to capture sequence videos of the ground, to gather remotely an accurate and real‐time view more broadly about the area of interest. Also, through the thermal camera mounted, the medical staff in the GCS can identify new cases without having to touch those who might be infected. It is equipped with sensor‐like cameras such as thermal cameras. Captured images/videos are mined to check if the public keeps social distancing, detect some COVID‐19 symptoms (see Figure 2) etc.. Moreover, UAVs can exchange relevant information with GCS and eventually broadcast it to the public (e.g., via street display screens, text/voice messages, etc.). This information can be: a) current status of COVID‐19 propagation; b) alert messages to sustain social distancing when barrier measures are broken; and c) reminders of the prevention techniques and best practices.

**FIGURE 3:**
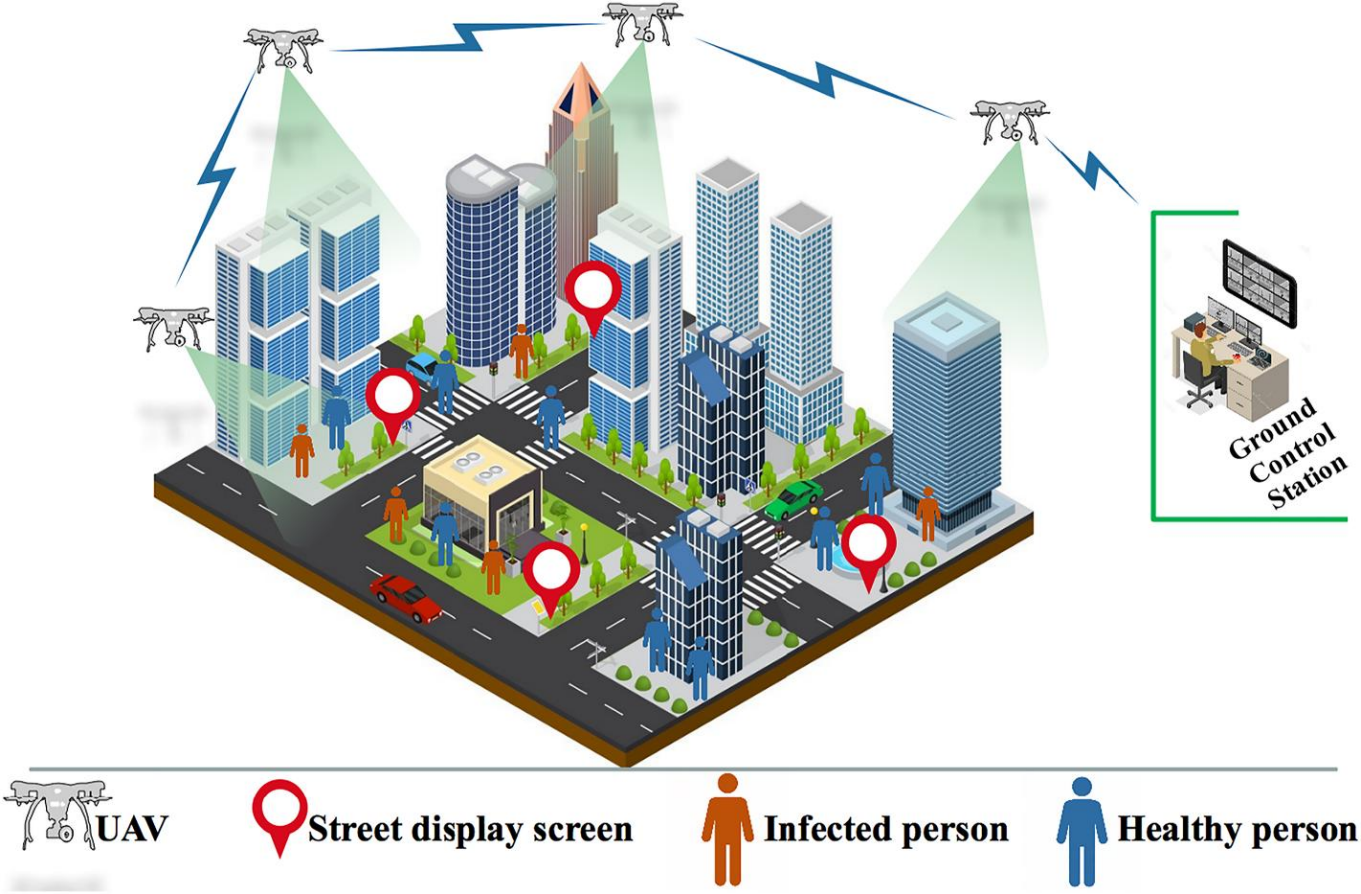
UAV‐augmented system to combat COVID‐19 outbreak, to force social distancing, spray disinfectants, broadcast messages, and deliver medical supplies

- **Target area:** Urban or sub‐urban areas;
- **UAV typology:** UAVs with rotary wings can hover over an area, fly at low altitudes to collect imagery and video in high resolution; The technology of Beyond Visual Line of Sight flight can be used as a type of drone that is used to protect drone operators in the event of a pandemic.
- **Number of UAVs:** In general, multi‐UAV operations are preferred to single‐UAV operations to cover a wide area collectively and to take videos from various vantage points.
- **Communication:** Multiple drones are operating in an ad hoc manner and the exchanging data is realized in multi‐hop UAV relaying as depicted in Figure 3;
- **Wireless communication technology:** Wi‐Fi standard;
- **Mobility pattern** We adopt path‐planned mobility model as it captures real application scenario traces [34];
- **Routing:** Static routing is more suitable for this use‐case.

Furthermore, managing latency for video streaming over drone networks is challenging. Indeed, the design planner must compile and co‐design a hardware/software solution offering below 150 ms latency.

### 5.2 COVID‐19 by design UAV‐assisted application

While this paper aims to design a scalable development process and tackle technical challenges, the major constraint facing the deployment of autonomous flying drones is legislation and some ethical considerations (see Figure 2).

#### 5.2.1 Unmanned traffic management (UTM) systems

Unsurprisingly, NAA, transportation departments and industry are developing UTM systems (https://utm.arc.nasa.gov). An UTM ecosystem aims to autonomously control operations of Unmanned Aerial Systems (UAS) for millions of commercial drones [35]. They are collaboratively exploring regulations, concepts of operation, data exchange requirements, and a supporting framework to enable multiple UAS operations beyond visual line‐of‐sight at altitudes under 400 ft above the ground level in airspace where NAA air traffic services are not provided. Some drone service providers, such as Wing (https://wing.com) and Zipline (https://flyzipline.com), have developed their own UTM software.

#### 5.2.2 Privacy and ethical considerations

During the COVID‐19‐pandemic, sensor data points, digital surveillance tools and health‐check applications were largely deployed to curb the disease spread [36]. However, how this data is cross‐checked and reused for surveillance purposes can easily exacerbate privacy and data‐protection concerns. Thus, digitally accessible data and algorithms for prediction and surveillance are of vital importance in combating the COVID‐19 pandemic, and it is equally necessary to (re)use them responsibly, in compliance with the regulations on data security and with proper respect for privacy and confidentiality.

## 6 SIMULATION RESULTS: COVID‐19 CASE

We use OPNET Modeller as a simulation environment to implement our UAVaaS scheme. Each drone is equipped with two antennas: a directional antenna to communicate with ground, while an omnidirectional antenna is used to communicate with the neighbouring drones. We consider a Longley‐Rice propagation model, which turns to be appropriate for urban areas, since it considers environmental conditions along the signal pathway (terrain, buildings, etc.); otherwise, it predicts radio field strength based on the elevation profile of the terrain between the transmitter and the receiver. Table 1 lists the main settings used in the simulation.

**TABLE 1:** Parameter settings

Parameter	Value
Area	2000 × 800^2^
UAV mobility model	Angular &linear
Data rate	54 mbps
Transmit power	100mW
Drone's receiver sensitivity	−95dBm
Beacon transmission interval	0.02s
Retry limit per packet	4

**FIGURE 4:**
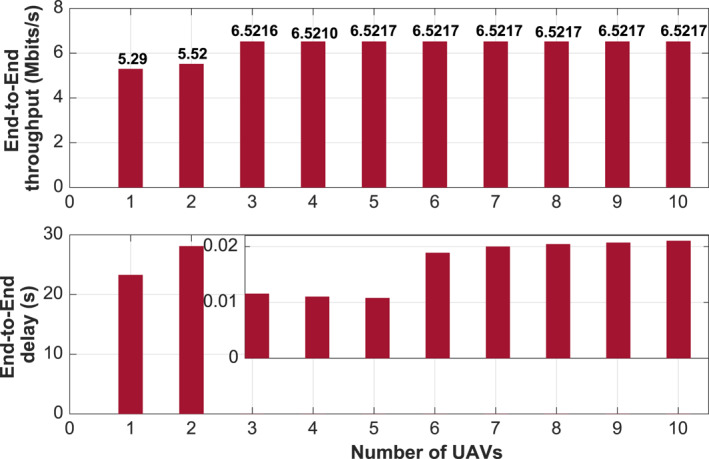
e2e Throughput and e2e delay versus the fleet size

**FIGURE 5:**
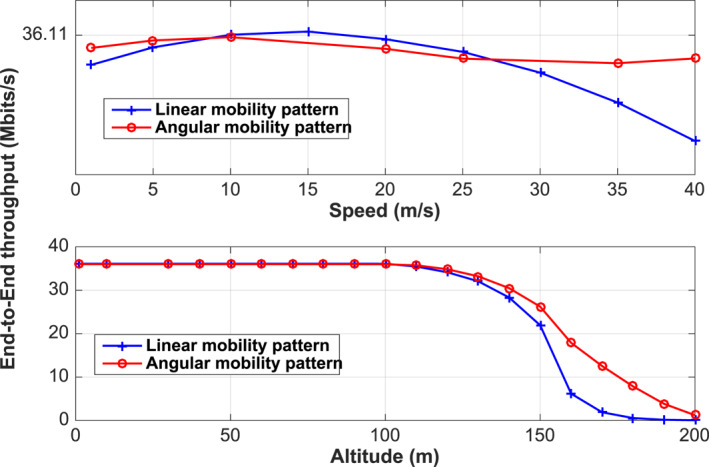
End‐to‐End delay versus Speed and Altitude

We first identify and characterise the terrain data corresponding to the geographic area where the application will be deployed. Topological design stage is of utmost importance; it aims to determine the optimal number of drones required to cover the target area (2000 × 800 m^2^). In order to assess how the multi‐hop network might suffer from inappropriate routing, we depict in Figure 4 the e2e throughput and the latency while varying the number of drones. Numerical results show that there exists an optimal number of UAVs that minimises the latency while the throughput remains constant. Increasing the number of UAVs increases the contact time/opportunity with GCS, which improves both the throughput and the latency. However, deploying further UAVs turns to have the reverse effect. Now, the UAVaaS coordinator has only to pick the UAV fleet size meeting the throughput/latency requirements. The UAV altitude and speed are the two, among others, design parameters that the design planner has to provide while designing the application. Figure 5 shows the impact of these two parameters on throughput and latency, while considering both linear mobility and angular mobility. We notice that there exists an optimal UAV speed that maximises the throughput under both mobility schemes. A special feature is that the angular mobility offers a stable throughput for any speed value. Meanwhile, throughput collapse can be observed under linear mobility at high speeds, which may affect the video smoothness. Figure 5 shows that the throughput remains stable for low altitude, that is, less than 100 m. However, the throughput vanishes rapidly when the altitude exceeds 150 m.

**FIGURE 6:**
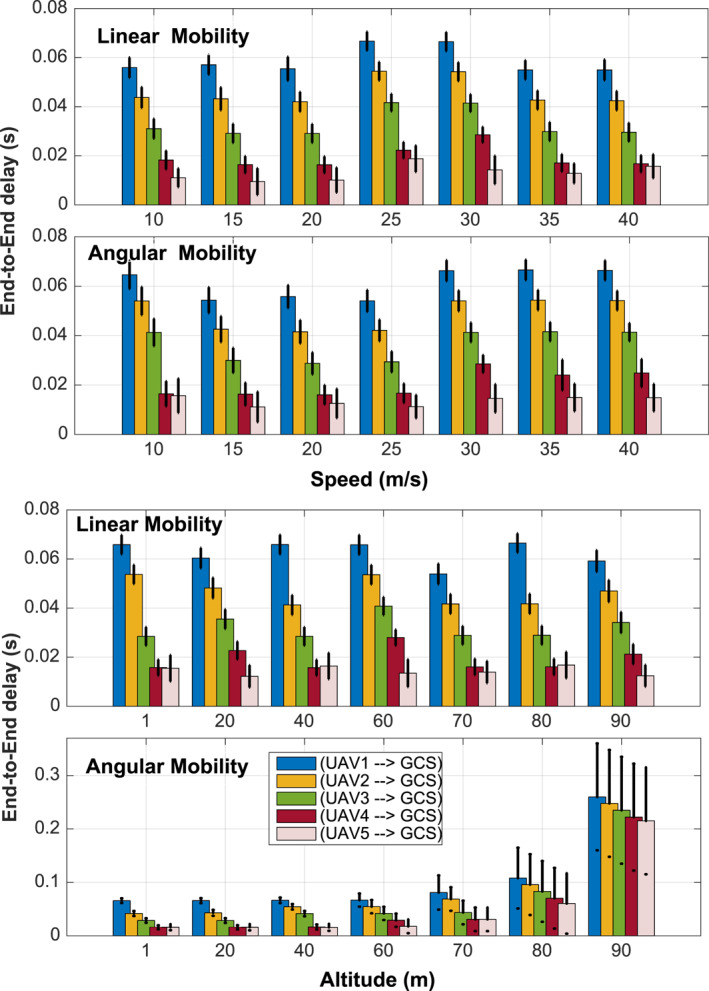
Impact of altitude (Up) and speed (Down) on end‐to‐end delay

Figure 6 depicts the effect of altitude and speed on the latency under different mobility patterns. Per stream latency is assessed while varying the speed/altitude. Linear trajectory seems to offer a lower latency compared to angular trajectory. It is worth noting that angular pattern allows to record videos from different viewpoints, which might be of utmost importance.

## 7 OPEN ISSUES

The network design is an extremely complex process, and many hot topics arise while dealing with UAV‐swarm networks. On the basis of the proposed framework for UAV as a service, there exist several open research issues that must be investigated for the suitability of designing UAV applications. The main challenges are highlighted in the following:

– **Devices rise**: The uncontrollable variability of traffic generated over time is linked to the heterogeneity and disparity of devices/users attached to the UAV‐network. This might generate unexpected traffic peaks hard to be handled. Traffic flow interferes the network to offer enough bandwidth to implemented applications per device class. Then, planning a UAV network supporting heterogeneous devices is still a hot topic to address in‐depth;
– **Reliability**: Due to an abnormal topology changes, such a network may experience failures at the node level or/and bottleneck link between a couple of source‐destination pairs. Providing an alternate path from a given source and a given destination via a set of UAV relays is a common solution to guarantee e2e reliability. However, the UAV’s extreme mobility and limited battery lifetime are challenging the efficiency of such a solution;
– **Connectivity**: Providing network access to drones and from/to other devices is crucial for a FANET to qualify as a communication network. UAVs that are deployed need to fly in the covered area to keep connected. Yet, the drone's limited battery is an obstacle ahead of a long‐term mission, which could lead to traffic congestion while having no access opportunity. Therefore, deciding between adding a new UAV to the UAV swarm or taking a drone (low‐battery) off is a question to address;
– **Safety:** Attacks against UAVs can trigger significant harm to the application. For instance, a civilian application might violate individual privacy by gathering personal information (photos, videos, position, etc.). “No‐fly‐zone” and tacking intruding/unauthorized UAVs are the two proposed defence approaches facing malicious use of UAV. However, a sthese techniques suffer from GPS spoofing attacks, obstructing these attacks is worth investigations;
– **Security:** The applications serving massive users and ultra‐dense areas are likely to eavesdrop unauthorized nodes on the ground and/or alter data over the UAV swarm. Authentication and cryptography, or enabling physical security via inherent features of wireless channels are considered as an efficient solutions for the network security [37]. Consequently, managing security aspects depends on the application itself, on the target environment and on the technology embedded aboard UAVs, while taking into account the attacker's potential motivations.

## 8 CONCLUSION

We propose a framework for UAV as a service. It incorporates all the actors/stakeholders contributing to the UAV application, and outlines their roles and describes how they interact. This roadmap aims to help planning, sizing and counting a UAV‐augmented application while meeting some target SLA. UAV‐assisted COVID‐19 surveillance and warning is proposed as a use‐case, where we showcase how to build the design planner, and provide insights on parameter settings in order to meet the SLA requirements.

## CONFLICT OF INTEREST

The author declares that there is no conflict of interest that could be perceived as prejudicing the impartiality of the research reported.

## Data Availability

The data used to support the findings of this study are available from the corresponding author.

## Footnotes

*
https://www.geospatialworld.net/blogs/what‐is‐bvlos‐and‐why‐is‐it‐important‐for‐drone‐industry
